# Enhanced Tribological Performance of Low-Friction Nanocomposite WSe_x_S_y_/NP-W Coatings Prepared by Reactive PLD

**DOI:** 10.3390/nano13061122

**Published:** 2023-03-21

**Authors:** Vyacheslav Fominski, Dmitry Fominski, Maxim Demin, Roman Romanov, Alexander Goikhman

**Affiliations:** 1 National Research Nuclear University MEPhI (Moscow Engineering Physics Institute), Kashirskoe sh. 31, 115409 Moscow, Russia; 2 Kant Baltic Federal University, A. Nevskogo St. 14, 236016 Kaliningrad, Russia

**Keywords:** reactive pulsed laser deposition, low-friction coatings, tribo-induced modification, nanoparticles, tungsten sulfoselenide, wear

## Abstract

A novel laser-based method for producing nanocomposite coatings consisting of a tungsten sulfoselenide (WSe_x_S_y_) matrix and W nanoparticles (NP-W) was developed. Pulsed laser ablation of WSe_2_ was carried out in H_2_S gas under appropriate laser fluence and reactive gas pressure. It was found that moderate sulfur doping (S/Se ~0.2–0.3) leads to significant improvement in the tribological properties of WSe_x_S_y_/NP-W coatings at room temperature. Changes in the coatings during tribotesting depended on the load on the counter body. The lowest coefficient of friction (~0.02) with a high wear resistance was observed in a N_2_ environment at an increased load (5 N), resulting from certain structural and chemical changes in the coatings. A tribofilm with a layered atomic packing was observed in the surface layer of the coating. The incorporation of nanoparticles into the coating increased its hardness, which may have influenced the formation of the tribofilm. The initial matrix composition, which had a higher content of chalcogen atoms ((Se + S)/W~2.6–3.5), was altered in the tribofilm to a composition close to the stoichiometric one ((Se + S)/W~1.9). W nanoparticles were ground and retained under the tribofilm, which impacted the effective contact area with the counter body. Changes in the tribotesting conditions—lowering the temperature in a N_2_ environment—resulted in considerable deterioration of the tribological properties of these coatings. Only coating with a higher S content that was obtained at increased H_2_S pressure exhibited remarkable wear resistance and a low coefficient of friction, measuring 0.06, even under complicated conditions.

## 1. Introduction

Transition Metal Dichalcogenide (TMD) coatings are highly regarded for their exceptional solid-lubricating properties. Research into TMD coatings has been ongoing for several decades, with a growing emphasis on exploring their properties [1,2,3,4,5]. The antifrictional (low-friction) properties of TMD coatings are attributed to their hexagonal crystal lattice structure, which features layered atomic packing. The weak shear resistance between the basal planes is widely considered the primary factor contributing to the solid-lubricating behavior of TMD-based coatings [5,6,7,8,9]. However, this view of the friction mechanism does not fully explain the tribological variability seen in TMD coatings of varying phases, their chemical composition, or architecture. As a result, there is continuing interest in investigating the ways to enhance the wear resistance of TMD coatings in a variety of conditions (such as medium composition and temperature) and to achieve ultralow friction [4,5,10,11,12,13,14,15].

The most widely utilized method for obtaining TMD-based coatings is ion sputtering, also known as magnetron deposition. This technique allows the composition of deposited coatings to be flexibly adjusted and the tribomechanical characteristics to be improved. This is achieved through forming multilayer or compound coatings with metals (Ti, Ni, Pb, etc.) to enhance their corrosion resistance and low-friction properties via intercalation [16,17,18]. Improved hardness and wear resistance have been achieved by alloying TMD-based coatings with carbon and/or nitrogen [15,19,20,21,22]. Another approach is to form multilayer coatings in which thin (nanometer) TMD layers alternate with carbon layers having different concentrations of diamond bonds between carbon atoms (a-C/DLC: amorphous/diamond-like carbon) [23,24]. This configuration allows for the control of TMD phase growth and the synergistic effect between a-C/DLC and TMD phases, leading to superlubricity [25,26,27,28,29]. To fabricate nanocomposite TMD-containing coatings, TMD and mono/multielement targets such as TiN or TiCrB are used for co-deposition by ion sputtering [30,31,32]. The phase composition/morphology of these coatings is determined by the thermodynamic properties of the selected element systems, the kinetic conditions depending on the deposition temperature, and ion bombardment intensity.

Pulsed laser deposition (PLD) is used more rarely in producing TMD-based coatings than magnetron deposition. Although initial experiments and subsequent studies have demonstrated the potential of PLD in obtaining TMD coatings, it remains limited due to the complexity of laser systems and the challenges in depositing coatings on larger surfaces. Yet, the unique features of PLD, such as versatility, ease of integration with ion irradiation, and the ability to control ion-plasma flux energy, offer new avenues for exploring the development of TMD coatings with improved characteristics [33,34,35,36,37]. One of the distinctive aspects of PLD in forming TMD-based coatings is the emission of submicron and nanometer-sized particles from TMD targets during pulsed laser ablation [33,37,38,39]. The concentration, size, structure, and chemical composition of these particles are influenced by both the target composition and the intensity of laser impact [38,40,41]. Previous studies have shown that the ablation of MoS_2_ and MoSe_2_ targets produces b.c.c. Mo nanoparticles [37,42]. Meanwhile, the ablation of WSe_2_ targets caused β-W nanoparticles (NP-W) to be deposited on the substrate surface [39]. In the cases of low particle density, the particles are embedded in the coating structure without a reduction in density [43]. Note that these results were obtained using yttrium aluminum garnet YAG(Nd) pulsed lasers, and the picture may change when using excimer lasers, where the emission of metallic nanoparticles is less efficient [35]. Two techniques for generating metal nanoparticles in TMD coatings using nanosecond laser pulses are currently being considered. The first one involves the selective or fractional evaporation of the TMD target to form vapor and liquid metal phases, with the liquid phase solidifying on the coating surface in the form of rounded particles [38,42,44]. The other technique involves the condensation of the laser plume to form metal nanoparticles during the target-to-substrate phase [45].

If the density of the metal nanoparticles is high, they can have a significant impact on the tribological properties of TMD-based coatings. On the one hand, these particles can cause accelerated abrasion of the coating; on the other hand, rounded particles can affect contact with the counter body. The contact area may change, or sliding friction may turn into rolling friction. To investigate this issue, in this study, WSe_2_-based coatings with an increased concentration of W nanoparticles were obtained, and their chemical state and structure were analyzed. The chemical composition of the coatings was modified by introducing sulfur atoms. Several previous studies have established for several coatings produced by magnetron and laser deposition that mixing different chalcogens (S and Se) has a positive effect on the tribological properties of TMD coatings [16,17,35,46]. The reactive PLD (RPLD) technique has been used to alloy the WSe_x_/NP-W coatings with sulfur. WSe_x_S_y_/NP-W coatings have been formed by pulsed laser ablation of the WSe_2_ target in hydrogen sulfide at different pressures. The tribological properties of WSe_x_S_y_/NP-W coatings with different S concentrations were investigated in wet air and a N_2_-enriched environment at room temperature. Additionally, this study presents the results of tribotests conducted at −100 °C in a N_2_-enriched environment, which demonstrated the superior performance of WSe_x_S_y_/NP-W coatings with a high concentration of chalcogen atoms under these complicated sliding conditions.

## 2. Materials and Methods

The WSe_2_ target for laser ablation was synthesized using a cold-pressing technique with WSe_2_ micropowder of 99.9% purity. The details of the target synthesis are described in a previous publication [39]. The target was ablated using the modulated Q-switched mode of a Solar LQ529 yttrium aluminum garnet laser (Minsk, Belarus) with a wavelength of 1064 nm. The laser emitted 15 ns pulses with a maximum energy of 80 mJ and a fluence of 9 J/cm^2^, with a pulse repetition rate of 20 Hz. The laser beam was directed at a 45-degree angle to the surface of the target, and an optical scanning system was used to move the beam across the surface. The silicon substrates, which were selected based on the favorable tribological properties of WSe_x_ coatings on Si-containing underlayers, found by Shtansky et al. [39], were positioned parallel to the target at 4 cm. During ablation, the target and substrate were placed in a chamber with air evacuated by a turbomolecular pump to achieve a pressure of ~10^−3^ Pa. To alloy the WSe_x_/NP-W coatings with sulfur, hydrogen sulfide gas was introduced into the chamber at pressures of 3.6 and 9 Pa. The obtained coatings were designated as WSe_x_S_y_/NP-W_3.6 and WSe_x_S_y_/NP-W_9, respectively. The substrate temperature was maintained at room temperature, and the pressure was selected based on prior studies on TMD film deposition by RPLD [47]. The deposition time for the coatings varied from 15 to 25 min.

The surface morphologies of the fabricated coatings and their chemical composition were studied by scanning electron microscopy and by energy-dispersive X-ray spectroscopy (SEM/EDS, Tescan, LYRA 3, Brno, Czech Republic). The crystal structure of the coatings was examined by grazing incidence X-ray diffraction (XRD) using an angle of 10° and Cu Ka radiation in an Ultima IV (Rigaku, Japan) diffractometer. The chemical states of the coatings were studied by X-ray photoelectron spectroscopy (XPS). The XPS spectra were obtained by a Theta Probe Thermo Fisher Scientific (Waltham, MA, USA) spectrometer with a monochromatic Al Kα X-ray source (1486.7 eV) and an X-ray spot size of 400 μm. The photoelectrons had a take-off angle of 50° with respect to the surface plane. The spectrometer energy scale was calibrated using Au4f_7/2_ core-level lines located at a binding energy of 84.0 eV. The structure of the coatings before and after tribotesting was studied by micro-Raman spectroscopy (MRS) using a 632.8 nm (He–Ne) laser; the laser-beam cross-section was less than 1 μm. The modes of MRS spectrum measurements (laser-beam intensity and measurement time) were selected in such a way that structural and chemical changes occured in the coatings.

The structure and composition of the coatings at the nanoscale were analyzed before and after tribological testing using scanning transmission electron microscopy (STEM) and energy dispersive spectroscopy (EDS) on an OSIRIS TEM/STEM instrument (Thermo Fisher Scientific, Waltham, MA, USA) equipped with a Bruker EDS detector. A thin cross-section of the WSe_x_S_y_/NP-W-coated silicon substrate was prepared using a focused ion beam and investigated by STEM with an accelerating voltage of 200 kV. The protective Pt/C film was applied before sample preparation for STEM studies. The acquired images were processed with the help of Digital Micrograph software (Gatan, USA) and TIA software (Thermo Fisher Scientific).

The indentation hardness of the films was determined using a Nano Hardness Tester (CSM Instruments, Switzerland) equipped with a Berkovich diamond indenter tip, calibrated against fused silica. The tribotesting of the thin-film coatings was carried out by an Anton Paar TRB3 tribometer in a reciprocating motion mode, using a steel ball (100Cr6) with a diameter of 6 mm as a counter body. The loads were 1 and 5 N. The sliding speed of the ball on the coated substrate was 2 cm/s. The tribotests were performed at 22 °C in air at a relative humidity of RH~35% and in a N_2_-enriched environment (RH~7%). In some cases, the tested Si plate was mounted in a holder cooled with liquid nitrogen. To prevent water vapor condensation and ice layer formation, the friction pair was treated in a N_2_-enriched environment. An optimal N_2_ flow rate made it possible to keep the plate temperature at about −100 °C and remove the air from the area surrounding the tested sample. The characteristics of the wear tracks on the coatings, the wear scar on the balls, and the wear debris were obtained using an optical profilometer and optical microscopy. After tribotesting, additional studies of the wear tracks by STEM, EDS, and MRS were carried out.

## 3. Results

### 3.1. Morphology, Composition, and Microstructure of WSe_x_/NP-W and WSe_x_S_y_/NP-W Coatings

**Figure 1:**
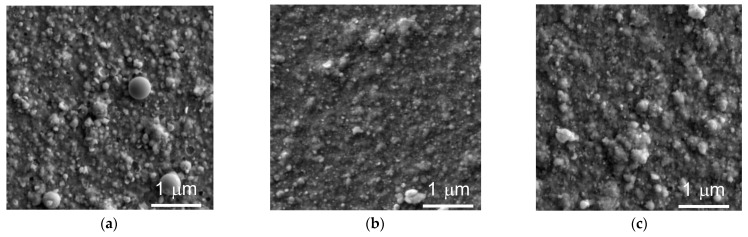
SEM images of the surface of coatings obtained by pulsed laser ablation of a WSe_2_ target (**a**) under vacuum condition and (**b**,**c**) in reactive H_2_S gas at pressures of 3.6 and 9 Pa, respectively.

Figure 1a–c shows SEM images of WSe_x_/NP-W and WSe_x_S_y_/NP-W coatings deposited on a substrate during 25 min. The coatings have a sufficiently dense structure, which is weakly dependent on the deposition conditions. The surface showed submicron rounded particles consisting of a cluster of smaller spherical nanoparticles. On the surface of the WSe_x_/NP-W coating, there was only a small number of spherical particles, with diameters of up to 0.5 µm.

The study of the WSe_2_ target subjected to pulsed laser ablation revealed the formation of rounded particles enriched in tungsten. The SEM, EDS, and XRD results of the laser-irradiated WSe_2_ target are shown in Figures S1–S3 in the Supplementary Materials. The XRD analysis demonstrated the emergence of two new phases in the target post irradiation, which consisted of cubic α-W and a metastable phase characterized as W_3_O and β-W. The stabilization of the β-W phase is attributed to the introduction of O (and potentially Se/S) atoms. The analysis of peak intensities suggests that the formation of α-W was predominant on the surface of the target.

The mechanism behind the modification of phase composition in a laser-ablated WSe_2_ target requires further investigation. This mechanism encompasses the formation of superheated liquid W-Se phase, potentially accompanied by the selective evaporation of selenium and the coagulation of the thin film of W-enriched liquid into spherical particles that then solidify. When the laser impacts a local target area, the particles are likely to be captured by the laser plume and transported to the coating surface. Large particles have low velocities and are unlikely to adhere to the coating. Smaller particles (nanoparticles) exhibit high velocities (~2 × 10^4^ cm/s) and are more likely to adhere to the coating surface [44].

**Figure 2:**
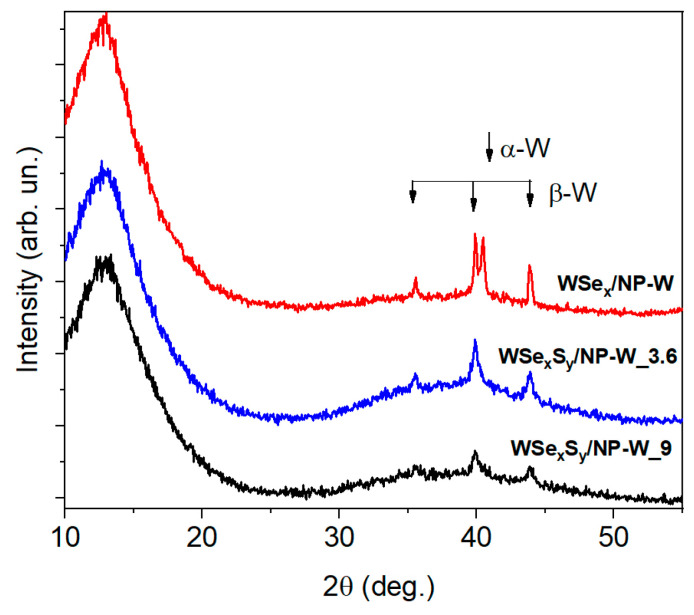
XRD patterns of WSe_x_/NP-W and WSe_x_S_y_/NP-W coatings obtained on Si substrates by PLD and RPLD at 3.6 and 9 Pa pressure of H_2_S gas.

XRD studies of the laser-deposited WSe_x_/NP-W coating confirmed the formation of a structure consisting of an X-ray amorphous matrix and a β-W/W_3_O phase (Figure 2). A detailed analysis of XRD data for coatings of this type was carried out earlier in [39]. Along with the β-W phase, the formation of the α-W phase was also found. The S atom penetration had no significant effect on the structure of the WSe_x_S_y_/NP-W coatings; however, the contribution from inclusions of the α-W phase in the XRD patterns for these coatings was noticeably smaller than for the WSe_x_/NP-W coatings. Perhaps, this is because S atoms are introduced into the molten/hot W particles transferred by the laser plume from the irradiated target to the coating and stabilize the β-W phase during their “quenching”.

EDS (SEM) elemental analysis showed that in a relatively large volume of WSe_x_/NP-W coating the x = Se/W ratio was 2.1, which was almost identical to the composition of the pristine target. For the WSe_x_S_y_/NP-W coating deposited with a H_2_S pressure of 3.6 Pa, the S/Se ratio was 0.5, and the (Se + S)/W ratio was 2.4. An increase in the H_2_S pressure to 9 Pa led to a further rise in sulfur content, resulting in a S/Se ratio of 2.3 and a (Se + S)/W ratio of 4.3. According to EDS (SEM) measurements, the oxygen concentration in WSe_x_/NP-W and WSe_x_S_y_/NP-W coatings was ~3–4 at.%. Considering that the particles in these coatings contain mainly tungsten, the amorphous matrix of these coatings would be significantly enriched in chalcogen atoms.

### 3.2. Nanostructure of WSe_x_S_y_/NP-W Coatings

**Figure 3:**
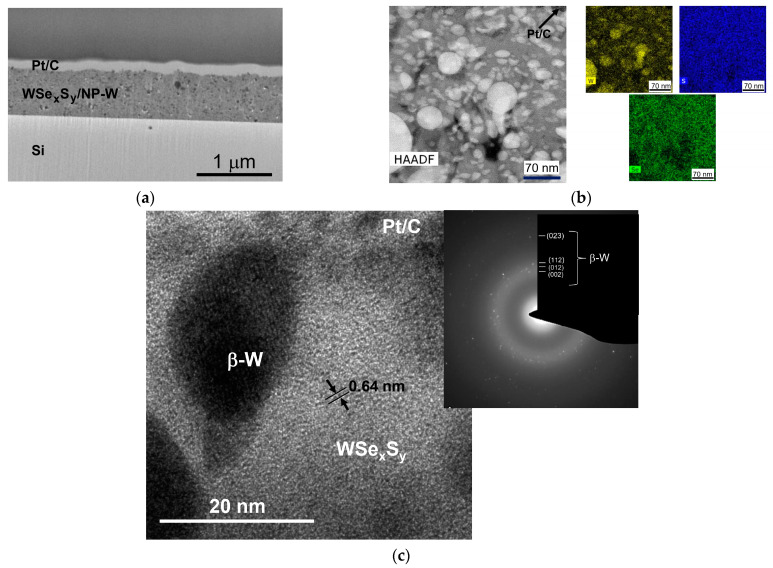
Cross-sectional STEM analysis of a WSe_x_S_y_/NP-W coating prepared by RPLD at a H_2_S pressure of 3.6 Pa on a Si substrate: (**a**) a low-magnification TEM image of the coating; (**b**) a high-angle annular dark-field (HAADF) STEM image of the surface layer of the coating (left) and colored EDS elemental maps of W, Se, and S in this local area (right); and (**c**) an HRTEM image of a local area in the surface layer. The inset contains the SAED pattern.

Figure 3a,b shows the results of a STEM study of a whole cross-section for WSe_x_S_y_/NP-W coating prepared at a 3.6 Pa pressure of reactive H_2_S gas for 15 min. The coating had a thickness of approximately 900 nm. The high-angle annular dark-field images and elemental maps reveal that the WSe_x_S_y_/NP-W coating was composed of a Se- and S-enriched matrix with embedded W-enriched nanoparticles. The coating contained regular, spherical particles with a diameter of up to 100 nm, in addition to particles of a deformed ellipsoid shape. The latter particles had a size of less than 50 nm. The density of the nanoparticles was high, while the coating exhibited a relatively dense structure with pores of up to 50 nm. Observations showed that the height of the surface protrusions on the coating did not exceed 100 nm. The analysis of high-resolution TEM (HRTEM) images and selected area electron diffraction (SAED) patterns confirmed the formation of nanoparticles with the characteristic crystal structure of the β-W phase (Figure 3c). The matrix exhibited an amorphous structure in which nanocrystals with layered atom packing were detected. These nanocrystals had a maximum size of 3 nm and a distance of 0.64 nm between the atomic planes.

The relatively high density of the WSe_x_S_y_/NP-W coating can be attributed to the high surface mobility of the atoms deposited from laser plume. The atoms move into shadow regions created by the deposition of spherical particles, leading to the formation of a relatively dense coating. However, this phenomenon may not occur when the deposited particles are molten and uniformly cover the coating surface. In such cases, the shape of the particle changes, as can be observed in the cross-sectional images of smaller particles.

EDS analysis was conducted on different local areas of the matrix, revealing a S/Se ratio of 0.2–0.3 and a (Se + S)/W ratio of approximately 2.6–3.5. The oxygen concentration did not exceed 6 at.%. A cross-sectional analysis of lager areas that included the NP-W showed a (Se + S)/W ratio of approximately 1.7, suggesting that the W atom content in the matrix and the NP-W was approximately equal. However, the EDS analysis of the nanoparticles indicated that they may also contain Se, S, and O atoms—possibly fragments of the matrix enriched with chalcogen atoms. With an estimated specific density of the amorphous matrix being 2–3 times lower than that of tungsten, the volume fraction of nanoparticles in the coating is estimated at 25–30%. The EDS elemental analysis of nanoparticles showed that they could contain Se, S, and O atoms, in addition to tungsten. Nevertheless, these results are not sufficiently reliable, as fragments of the matrix enriched with chalcogen atoms may have entered the measurement region.

**Figure 4:**
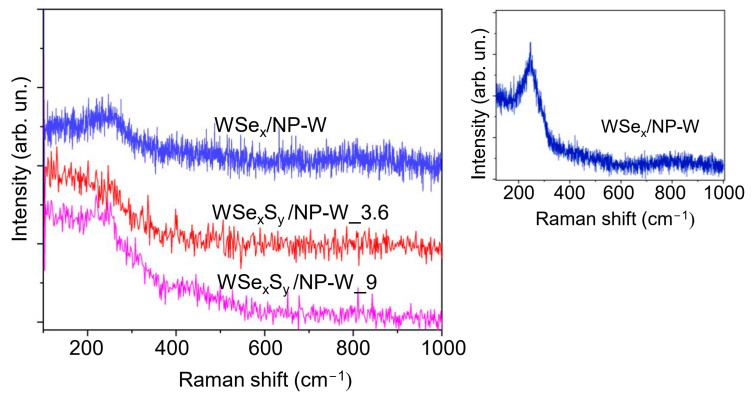
Raman spectra of WSe_x_/NP-W and WSe_x_S_y_/NP-W coatings measured with a laser intensity of 2 mW. The inset demonstrates that the use of laser irradiation with an intensity of 5 mW caused structure modification of the coating.

Figure 4 shows MRS spectra measured for WSe_x_/NP-W and WSe_x_S_y_/NP-W coatings. The spectrum of WSe_x_/NP-W coatings is in many ways like that obtained earlier for WSe_x_-based coatings deposited by PLD and magnetron techniques [48,49]. All spectra demonstrated a broad feature between 200 and 300 cm^−1^, which is characteristic of an amorphous/disordered local structure. The introduction of sulfur reduced the intensity of this broad peak, indicating an increased degree of local packing defectiveness in WSe_x_S_y_/NP-W films. The obtained films are in a highly nonequilibrium state, which can be easily altered by mild heating using a high-intensity laser beam. Upon laser heating, a narrower peak at approximately 250 cm^−1^ emerges (as shown in Figure 4). This peak may comprise two peaks (E^1^ _2g_ and A_1g_) from the WSe_2_ compound. Nevertheless, peaks related to various Se molecular configurations are also present in this frequency range [50,51]. Thus, during the heat-induced crystallization of the matrix in the WSe_x_/NP-W coating, surplus selenium may be distributed along the interstitials of the 2H-WSe_2_ lattice. Other changes, such as the formation of elemental Se inclusions or sublimation/evaporation of excess selenium, are also possible.

### 3.3. Chemical States of WSe_x_/NP-W and WSe_x_S_y_/NP-W Coatings

**Figure 5:**
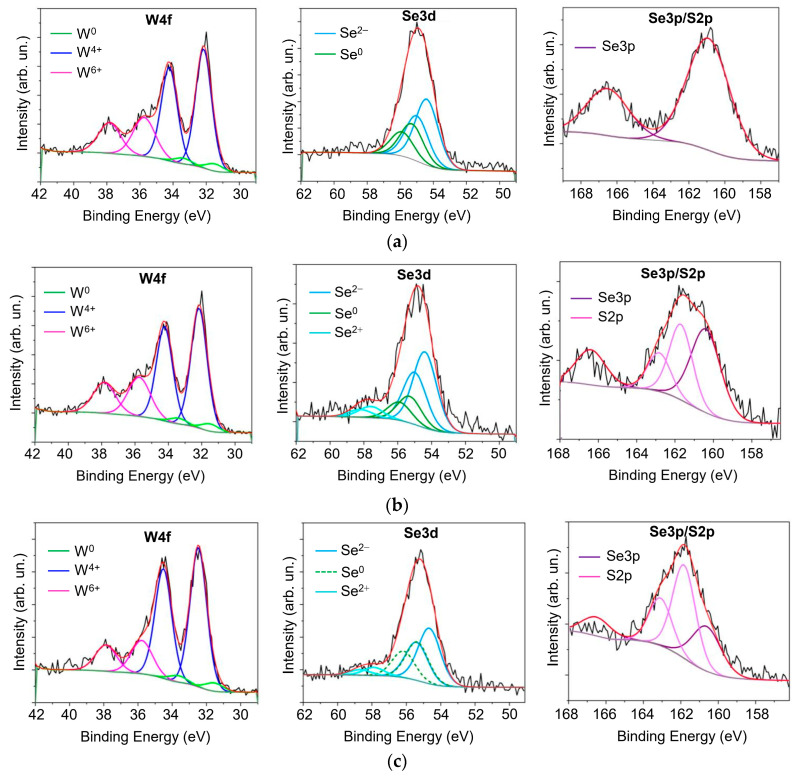
X-ray photoelectron spectroscopy scans of the (**a**) WSe_x_/NP-W, (**b**) WSe_x_S_y_/NP-W_3.6, and (**c**) WSe_x_S_y_/NP-W_9 coatings for the W, Se, and S elements.

Figure 5a–c shows XPS spectra measured for as-deposited WSe_x_/NP-W and WSe_x_S_y_/NP-W coatings. The analysis of the W4f spectra reveals the presence of W^0^, W^4+^, and W^6+^ species [52,53,54]. The dominance of W^4+^ and W^6+^ states is due to tungsten’s chemical bonds with chalcogen atoms (WSe_2_/WS_2_) and oxygen (WO_3_), respectively. An increase in sulfur concentration results in a shift of the W4f doublet peaks. The peaks for W4f_7/2_ and W4f_5/2_ in the WSe_x_/NP-W coating had binding energies of 32.16 and 34.23 eV, respectively. For the WSe_x_S_y_/NP-W_9 coating, these peaks were located at slightly higher binding energies of 32.47 and 34.52 eV. This energy shift and its increase with growing S concentration is a typical feature observed in metal diselenides upon partial replacement of Se atoms with S atoms [13,35,55]. The negligible contribution of the states of W^0^ to the total spectrum indicated that the W nanoparticles were effectively enveloped by the matrix material, even on the surface. It is possible that the W nanoparticles located on the coating surface oxidized, leading to relatively intense peaks in the XPS spectra due to W^6+^. The binding energies of the W4f_7/2_ and W4f_5/2_ peaks caused by metal oxide inclusions were almost independent of the coating composition and were found to be approximately 35.7 and 37.8 eV, respectively.

The XPS spectra of the WSe_x_S_y_/NP-W coatings reveal the presence of S2p peaks, along with Se3p peaks in the background. The Se3p peaks were analyzed using information obtained from the Se3p spectra of the pure WSe_x_/NP-W coating. The two peaks at 161.8 and 163.0 eV shown in the XPS spectra of the WSe_x_S_y_/NP-W coatings can be assigned to S2p_3/2_ and S2p_1/2_ binding energies, respectively [13,35]. The binding energies are independent of the S concentration. In addition, two peaks assigned to Se2p_3/2_ and Se2p_1/2_ were observed at 160.9 and 166.6 eV, respectively.

When analyzing the Se3d region in the XPS spectrum of pure WSe_x_/NP-W coating, the overstoichiometric composition of the matrix was considered. Two pairs of peaks were necessary to fit each Se peak, corresponding to Se^2−^ species located at different nodes in the amorphous mesh of the WSe_x_ matrix and to Se^0^ species in Se-enriched clusters [35,49,54,56].

The binding energies of the Se3d_5/2_ peak for these Se species are 54.4 and 55.3 eV, respectively. The S incorporation in the WSe_x_S_y_ matrix slightly increases the binding energy for the Se^2−^ peak and introduces a broad band at higher binding energies (over 57 eV), which could be due to the introduction of O and/or S atoms into the Se clusters. Surface oxidation of the coatings caused by prolonged exposure to air after coating deposition may also influence the chemical state of tungsten and selenium. The best fit of the experimental Se3d spectrum was obtained when the Se3d_5/2_ peak was at 57.8 eV, which corresponds to Se^2+^ species. Se-enriched clusters may form due to excess Se atoms segregating. During the formation of the WSe_x_S_y_/NP-W coatings by reactive PLD, Se and S atoms were embedded into the coating matrix through different mechanisms. Se atoms were introduced through physical vapor-phase deposition, while S atoms were probably introduced through chemical interaction with surface coating atoms. High chalcogen concentrations in the WSe_x_S_y_ matrix may result in Se phase segregation, as observed in previous studies of PLD processes in Se-based TMD films [48,56].

### 3.4. Frictional Behavior of WSe_x_/NP-W and WSe_x_S_y_/NP-W Coatings

**Figure 6:**
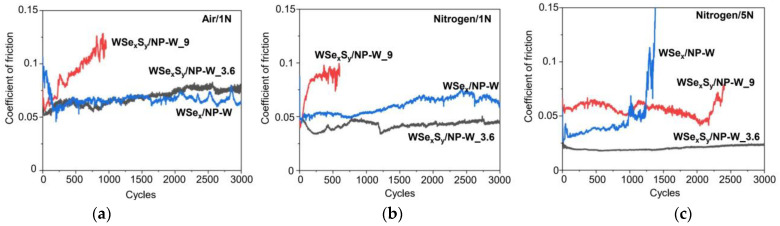
The friction coefficient of WSe_x_/NP-W, WSe_x_S_y_/NP-W_3.6, and WSe_x_S_y_/NP-W_9 coatings measured at room temperature in (**a**) air under load of 1N and N_2_-enriched environment under load of (**b**) 1N and (**c**) 5N.

Figure 6a–c shows the low-friction properties of WSe_x_/NP-W and WSe_x_S_y_/NP-W coatings at room temperature in environments consisting of moist air and a N_2_-enriched environment under loads of 1 and 5 N. The coating deposition time was 25 min. For the WSe_x_/NP-W coating, measurements in the air under a load of 1 N exhibited a behavior characterized by an initially higher coefficient of friction (CoF~0.09), which transitioned (run-in) to a lower steady-state CoF~0.07 with increased sliding cycles. This run-in behavior changed when this coating was doped with sulfur. For the WSe_x_S_y_/NP-W_3.6 coating, the initial CoF value was 0.05. During the run-in phase, the CoF increased to 0.06 and then slowly increased to 0.07 by the end of the test. For the WSe_x_S_y_/NP-W_9 coating, the CoF increased relatively quickly from an initial value of 0.06 to 0.12 when tested for 1000 cycles. The testing of this coating was then stopped.

The tribotests in a N_2_-enriched environment with loads of 1 N showed only a slight improvement in the friction properties of the WSe_x_/NP-W coating, particularly during the running-in phase, where no increased coefficient of friction was observed. Throughout the tribotesting, the steady-state CoF was ~0.05–0.06. In contrast, the addition of sulfur considerably improved the low-friction properties of the WSe_x_S_y_/NP-W_3.6 coating in this environment, resulting in a steady-state CoF of ~0.045. Changing the test environment did not improve the tribological properties of the WSe_x_S_y_/NP-W_9 coating.

Increasing the load on the counter body had a different effect on the tribological behavior of the WSe_x_/NP-W and WSe_x_S_y_/NP-W coatings when tested in a N_2_-enriched environment. For WSe_x_/NP-W, the CoF value decreased to 0.03–0.04, but a sharp increase in CoF was observed at a certain stage of the tribotest. The durability of the coating did not exceed ~1500–2000 sliding cycles. This behavior was repeated in three tribotests. The best tribological properties were found for WSe_x_S_y_/NP-W_3.6, with a CoF value of 0.02–0.025 throughout the entire tribotest period. Increasing the load caused some improvement in the tribological properties of the WSe_x_S_y_/NP-W_9 coating. During a sufficiently long sliding period (up to 2500 cycles), the CoF value did not exceed 0.06.

**Figure 7:**
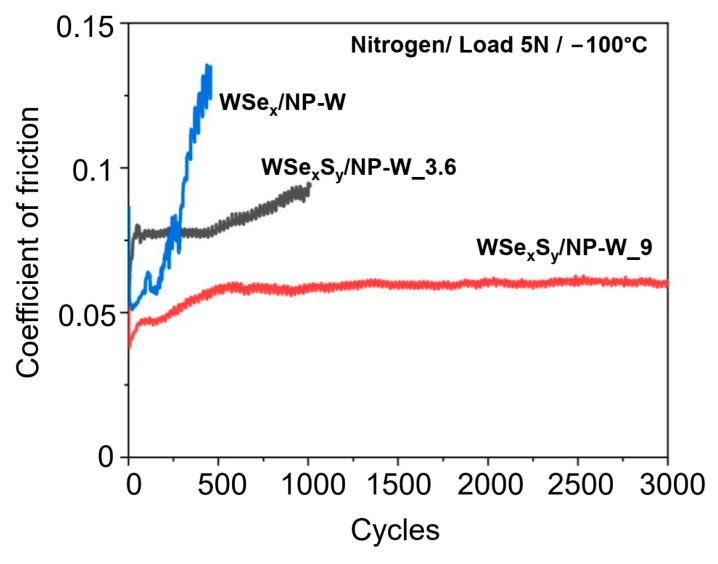
The friction coefficient of WSe_x_/NP-W, WSe_x_S_y_/NP-W_3.6, and WSe_x_S_y_/NP-W_9 coatings measured in a N_2_-enriched environment under a load of 5N. The tested samples were cooled to −100 °C.

The results of the tribotesting of the WSe_x_S_y_/NP-W_9 coating indicate that coatings with a high concentration of chalcogen atoms had unsatisfactory tribological properties (Figure 6). However, under specific conditions of low temperature and in a N_2_-enrichment environment, this coating showed the best tribological behavior (Figure 7). In these conditions, weak water vapor condensation and the formation of a thin ice film were possible. The WSe_x_S_y_/NP-W_9 coating had an initial CoF of 0.04 and reached a steady-state CoF~0.06 after 500 cycles. The CoF of the other two coatings increased rapidly to values above 0.08 within 500 to 1000 cycles.

The introduction of sulfur into the WSe_x_/NP-W coating caused a decrease in hardness and elasticity. The hardness of the WSe_x_/NP-W coating was approximately 9.2 GPa, i.e., higher than the 2–3 GPa hardness of magnetron-deposited WSe_x_ coating (e.g., [32,49]). Young’s modulus of the WSe_x_/NP-W coating was found to be between 110–120 GPa, which is significantly higher than that of magnetron-deposited WSe_x_ coatings, where Young’s modulus typically does not exceed 60 GPa [32,49]. The hardness of the WSe_x_S_y_/NP-W_3.6 and WSe_x_S_y_/NP-W_9 coatings was reduced to 7.5 and 4.3 GPa, respectively, due to the matrix modification. Young’s modulus of the WSe_x_S_y_/NP-W_3.6 and WSe_x_S_y_/NP-W_9 coatings was 107 and 67 GPa, respectively. Thus, the addition of S to the matrix of nanocomposite WSe_x_S_y_/NP-W coatings caused a decrease in their mechanical strength. However, the W nanoparticles present in the bulk prevented excessive softening of the coatings.

### 3.5. Wear Behavior of WSe_x_/NP-W and WSe_x_S_y_/NP-W Coatings

Studies into the tribological properties of WSe_x_/NP-W and WSe_x_S_y_/NP-W coatings have revealed that a comparison of the wear behavior of WSe_x_/NP-W and WSe_x_S_y_/NP-W_3.6 coatings is of particular interest under conventional test conditions (as regards ambient temperature and environment). The wear investigation focused on the central area of the track, as there was no significant correlation between wear and ball sliding speed in different areas of the track. Under specific test conditions, such as low temperatures and an environment containing water vapor, the sliding properties of these coatings were considerably impaired compared with the WSe_x_S_y_/NP-W_9 coating. Additional investigations are needed to determine the underlying reasons for this behavior. The Supplementary Materials (Figure S4) provide information solely on the ball scars and wear tracks that resulted from severe stress testing of these coatings, with a comparison with the wear tracks and scars of the balls for other prepared coatings.

**Figure 8:**
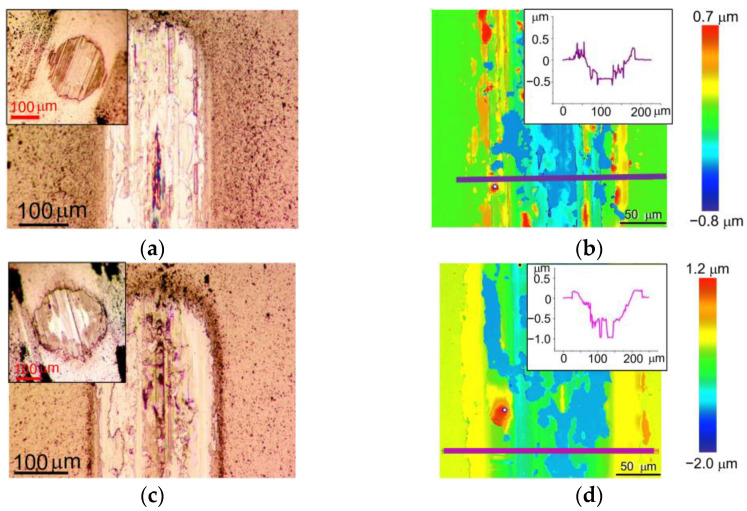
Wear of the (**a**,**b**) WSe_x_/NP-W and (**c**,**d**) WSe_x_S_y_/NP-W_3.6 coatings after tribotest in air at a load of 1 N: inserts in (**a**,**c**) show the wear scars of counter bodies (balls), and the depth profile of the wear tracks are shown in the inserts in (**b**,**d**).

Figure 8 shows the wear of the WSe_x_/NP-W and WSe_x_S_y_/NP-W_3.6 coatings and their corresponding counter bodies after tribotests in air. The coatings were deposited for 25 min. Notably, a correlation between the frictional and wear behavior of these coatings can be observed. The WSe_x_S_y_/NP-W_3.6 coatings and counter bodies experienced slightly higher wear than their WSe_x_S_y_/NP-W counterparts, resulting in a wider and deeper wear track and increased counter-body wear scar size. The depth profile of the wear track on the WSe_x_S_y_/NP-W_3.6 coating differed slightly from that of the WSe_x_/NP-W coating, with the former exhibiting large areas where the coating was completely removed from the substrate. The sharp edges of these areas suggest that the WSe_x_S_y_/NP-W_3.6 coating may have peeled off in relatively large fragments, whereas track deepening due to wear and tear on the coating was less prominent.

The weaker adhesion of the WSe_x_S_y_/NP-W_3.6 coating to the substrate compared with the WSe_x_/NP-W coating could be attributed to the decreased energy of deposited atoms and ions resulting from deposition in a reaction gas. This decrease in energy reduces the efficiency of chemical bond formation at the coating–substrate interface, and in turn, the efficiency of surface sputtering [41]. As a result of the suppression of self-sputtering and S atom deposition, the thickness of the coating formed by RPLD increased, with the WSe_x_/NP-W coating measuring 800 nm and the WSe_x_S_y_/NP-W_3.6 coating 1200 nm.

**Figure 9:**
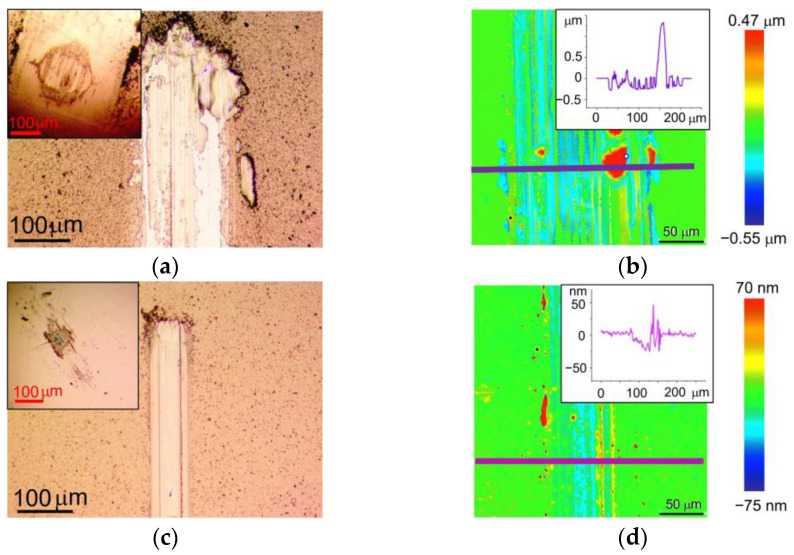
Wear of the (**a**,**b**) WSe_x_/NP-W and (**c**,**d**) WSe_x_S_y_/NP-W_3.6 coatings after tribotest in N_2_-enriched environment at a load of 1 N: inserts in (**a**,**c**) show the wear scars of counter bodies (balls), and the depth profile of the wear tracks are shown in the inserts in (**b**,**d**).

Figure 9 shows the wear of the WSe_x_/NP-W and WSe_x_S_y_/NP-W_3.6 coatings and counter bodies after the tribotests in a N_2_-enriched environment under a load of 1 N. The WSe_x_S_y_/NP-W_3.6 coating showed clearly improved wear resistance compared with the WSe_x_/NP-W coating. At the same time, the wear of the counter body was significantly reduced. The addition of S caused a reduction in track width from 150 µm to 80 µm. The maximum track depth reduced from 240 to 30 nm by a factor of 8. A comparison of the wear of these coatings in wet air and nitrogen showed that in N_2_ environment the wear decreased for both coatings. This behavior is typical of all TMD-based coatings. However, for WSe_x_S_y_/NP-W_3.6 coating, this reduction was the most significant.

**Figure 10:**
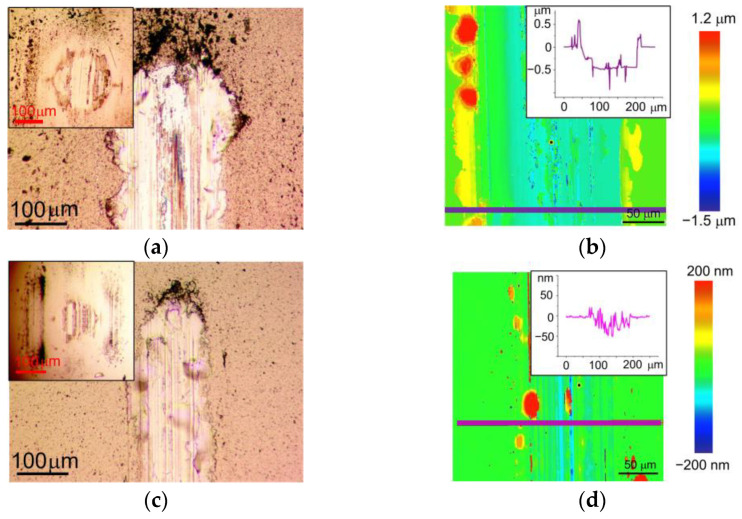
Wear of the (**a**,**b**) WSe_x_/NP-W and (**c**,**d**) WSe_x_S_y_/NP-W_3.6 coatings after tribotest in N_2_-enriched environment at a load of 5 N: inserts in (**a**,**c**) show the wear scars of counter bodies, and the depth profile of the wear tracks are shown in the inserts in (**b**,**d**).

During tribotesting of the WSe_x_/NP-W coating in N_2_-enriched environment with an increased counter body load of 5 N (Hertzian contact stress ~0.86 GPa), a marked increase in wear was observed. Figure 10 illustrates the wear pattern of the coating under these conditions, where the track depth on the coating reached nearly 500 nm after 1500 sliding cycles. The tribotest ceased upon a rapid escalation of the friction coefficient. The analysis of the wear track suggested that the cause of this phenomenon may be attributed to the localized deepening of the track down to the substrate. While the increase in load accelerated the wear of the WSe_x_S_y_/NP-W_3.6 coating, the increment was moderate. The average depth of the track rose to 40 nm, with some regions of the track exhibiting a wear depth increase to 100 nm. The width of the well also increased from 80 nm to 140 nm, with a concomitant growth in the wear scar of the ball. Notably, the exposure of the counter body to a high load resulted in the delamination of the coatings from the substrate in some localized areas of the track, as evidenced by the formation of visible swellings in the microphotographs of the tracks (Figure 9 and Figure 10). Wear analysis of the WSe_x_S_y_/NP-W_3.6 coating through the integration of the volume of material removed from the track demonstrated that the wear rate of this coating in a N_2_-enriched environment was independent of counter-body load, being registered at 1.6 × 10^−7^ mm^3^ N^−1^m^−1^.

When tested in a N_2_-enriched environment at a very low temperature, the WSe_x_S_y_/NP-W_9 coating with high sulfur content showed the least wear. After 3000 sliding cycles, a smooth track with minimal wear debris formed on the surface of the coating. Relatively low wear and a weak adherence of wear debris were also observed on the counter body. In the other coatings, the analysis of the wear tracks and the counter bodies showed that the rapid increase in the coefficient of friction, as seen in Figure 7, was due to the significant wear of the coatings and the counter bodies (Figure S4).

## 4. Discussion

**Figure 11:**
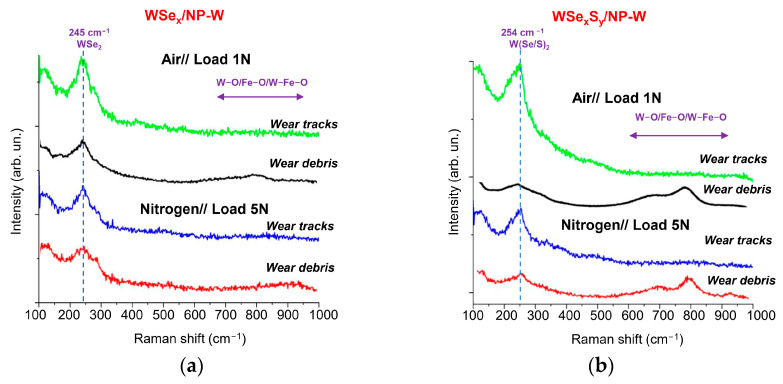
Raman spectra of the (**a**) WSe_x_/NP-W and (**b**) WSe_x_S_y_/NP-W_3.6 coatings measured after the tribotesting in air and N_2_-enriched environment for the center area of wear tracks and the wear debris located near the wear tracks.

The WSe_x_S_y_/NP-W_3.6 coating has tribological properties comparable to those of the WSe_x_/NP-W coating in moist air; it outperforms the WSe_x_/NP-W coating when tested in a N_2_-enriched environment at room temperature. Therefore, a detailed investigation of tribo-induced modification of the WSe_x_S_y_/NP-W_3.6 coating was carried out. Figure 11a,b shows Raman spectra for the wear tracks and wear debris formed after tribotesting the WSe_x_/NP-W and WSe_x_S_y_/NP-W_3.6 coatings. Under both test conditions, the coatings exhibited similar wear patterns, as indicated by the appearance of a relatively narrow peak near 250 cm^−1^, which is characteristic of the atomic packing of 2H-WSe_2_. However, the main difference in the Raman spectra measured in the wear tracks for the WSe_x_/NP-W and WSe_x_S_y_/NP-W_3.6 coatings was the shape of the peaks. In the spectrum of the WSe_x_/NP-W coating, the maximum peak was in the range of 235–245 cm^−1^. This shift from 250 cm^−1^ towards lower frequencies may be attributed to the influence of excess selenium, which can cause peaks at approximately 235 cm^−1^ in some nanoforms [57]. In contrast, the spectrum of the WSe_x_S_y_/NP-W_3.6 coating showed a peak maximum at 252 cm^−1^ and an asymmetrical peak shape. The shift towards higher frequencies was likely caused by the introduction of S atoms into the WSe_2_ lattice [58]. The asymmetry of the peak shape may be due to differences in the texture of the modified layers in these coatings, as the texture can affect the ratio of intensities of A_1g_ and E^1^_2g_ peaks, which merge into one broadened peak at a high defectiveness of atomic packing. Additionally, the formation of a structure containing WSe_2_ and W(Se/S)_2_ nanoclusters cannot be ruled out.

Raman studies of wear debris showed that, in addition to the S/Se-containing phase, there was a metal oxide phase as well (Figure 11a,b). The formation of metal oxide inclusions caused the appearance of a wide band from 700 to 1000 cm^−1^. The broad band extending from 600 to 800 cm^−1^ is situated in the region of W–O–W stretching bonds of WO_3_ [49,59]. The presence of shoulders extending over 700 and 900 cm^−1^ indicated a high degree of WO_3_-phase defectiveness and points to the possible formation of Fe-containing oxides (FeO, Fe_3_O_4_, and FeWO_4_) [60]. It is important to note that the N_2_-enriched environment reduced the influence of oxygen and water vapor on tribo-chemical processes in the friction zone but did not eliminate it. This phenomenon is more pronounced for the S-containing WSe_x_S_y_/NP-W coating as compared with pure WSe_x_/NP-W coating.

**Figure 12:**
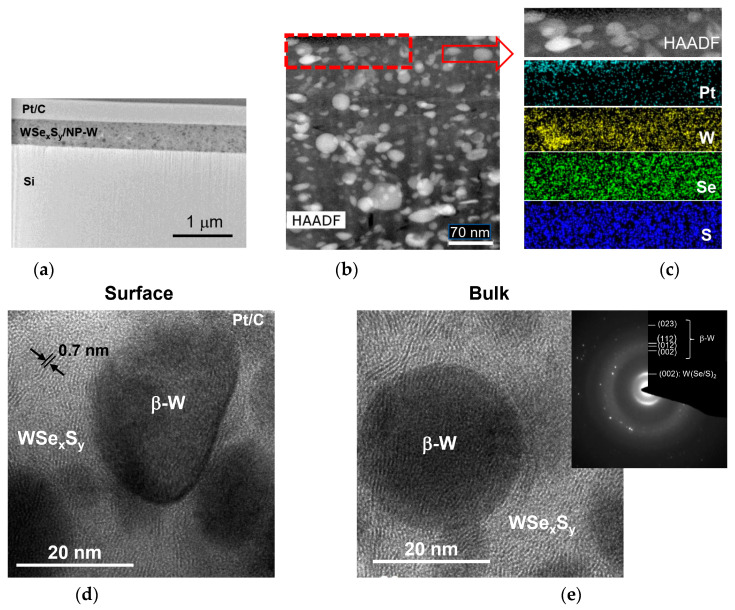
Cross-section STEM и SAED patterns of the WSe_x_S_y_/NP-W_3.6 coating subjected to tribotesting (500 cycles) in wet air at 1 N load on the counter body: (**a**) low-magnification TEM image of the coating; (**b**) HAADF STEM image of the coating; (**c**) the elemental maps obtained for a surface layer indicated in (**b**); and (**d**,**e**) HRTEM images obtained for (**d**) the surface and (**e**) deeper layers of the coating. The insert contains the SAED pattern which confirms the crystallization of the coating matrix.

Results of the STEM analysis of the WSe_x_S_y_/NP-W coating in the central region of the wear track formed after 500 sliding cycles in a humid air environment are presented in Figure 12a–e. The application of a 1 N load during sliding smoothed the roughness of the coating surface, while its porosity was retained. HRTEM and SAED studies revealed significant structural changes in the matrix throughout the coating, resulting in the formation of nanocrystals with a layered WSe_2_-like structure. The dimensions of the ordered areas with layered packing of atoms reached up to 20 nm, and their orientation was mainly influenced by the nearest W nanoparticle. The predominant orientation of the basic planes was along the surface of the nanoparticle, potentially due to the movement or vibration of the nanoparticle within the amorphous matrix. The interplanar spacing varied between 0.65 to 0.7 nm. No evidence of tribofilm formation with WSe_2_ nanocrystals oriented along the surface was found, nor was any change in the Se/S and (Se + S)/W ratios in the near-surface layer of the matrix detected. Exposure to moist air resulted in an increase in the O atoms concentration in the matrix surface layer to 11.7 at.%, with O atoms penetrating to a depth of 30 nm.

**Figure 13:**
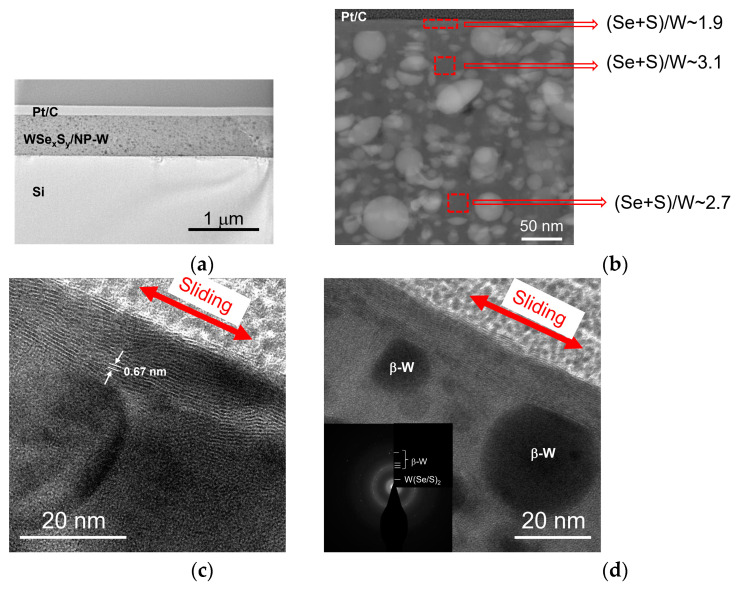
Cross-section STEM и SAED patterns of the WSe_x_S_y_/NP-W_3.6 coating subjected to tribotesting (~1000 cycles) in N_2_-enriched environment at 5 N load on the counter body: (**a**) low-magnification TEM image of the coating; (**b**) HAADF STEM image of the coating and EDS data of the local area composition; and HRTEM images exhibit (**c**) the layered tribofilm formation and (**d**) the wear of nanoparticles on the surface. The insert in (**d**) contains the SAED pattern for the surface layer.

Increasing the counter-body load to 5 N resulted in noticeable compaction of the WSe_x_S_y_/NP-W_3.6 coating and the effective formation of a W(Se/S)_2_-based tribofilm on the surface. Figure 13a–d presents HRTEM images and SAED patterns of the cross-section of a tribotested film in a N_2_-enriched environment after 1000 sliding cycles. The thickness of the tribofilm possessing ordered W(Se/S)_2_ fringes reached 15 nm, and the basic planes had a preferential orientation parallel to the coating surface. The distance between the basic planes was approximately 6.7 nm. At the boundary between the film and the matrix, the atomic planes exhibited arbitrary orientations. The thickness of such a transition layer did not exceed 10 nm. In deeper layers, the amorphous matrix structure was mostly preserved. The tribo-induced modification of the surface-layer structure involved the partial removal of S and Se atoms, resulting in a decrease in the (Se + S)/W ratio to ~1.9. However, the O atoms’ concentration did not change significantly in the surface layer after tribotesting.

Raising the load to 5 N did not lead to an effective crystallization of the matrix in the coating volume, as was observed in the lower load tests. This lack of structural transformation could be due to the strong compaction of the coating induced by a higher load, which may have inhibited the reduction in local atomic packing density required for the transformation of an amorphous structure into a layered one. However, the transformation of an amorphous matrix with an excessive content of chalcogen atoms ((Se + S)/W ~2.6–3.8) into crystalline 2H-W(Se/S)_2_ can cause the intercalation effects that increase the interplanar distance. This is evident from the increase in the base-plane spacing to 0.7 nm, as shown in Figure 12. Despite this, the formation of nanoclusters of these components cannot be excluded, although they were not detected in the original coating structure during XPS studies. Assuming that the tribo-induced structural changes in the WSe_x_S_y_/NP-W_3.3 coating in a N_2_-enriched environment at a load of 1N occurred in a similar way, the matrix crystallization throughout the coating can provide sufficient low-friction properties, even at a high concentration of chalcogen atoms—(Se + S)/W ~2.6–3.8. However, the results of tests under a higher load show that the densification of the structure and the formation of a tribofilm with a composition close to stoichiometric by the (Se + S)/W parameter are more important. The mechanisms responsible for removing excess S and Se atoms require further investigation. These atoms may be removed through the formation of volatile compounds with oxygen and hydrogen present in the applied media, such as wet air and a N_2_-enriched environment with an RH~7 %.

The analysis of the behavior of W nanoparticles in the WSe_x_S_y_/NP-W_3.3 coating revealed that these nanoparticles did not migrate into the coating and were removed during wear with the matrix material, under both high and low loads. This was confirmed by the consistent (Se + S)/W ~1.7 values measured by EDS in the cross-section of the coating. Yet, under lower loads, the nanoparticles were found to protrude above the coating surface, resulting in increased friction (Figure 12). Under higher loads, the nanoparticles were found underneath the tribofilm. Figure 13 shows that the rounded particles on the surface were ground because of contact with the counter body. The presence of a W(Se/S)_2_ tribofilm at the interface of the W nanoparticles and the Fe-based ball may induce tribo-chemical reactions, such as sulfurization and/or selenization of the nanoparticles and the steel ball, leading to phase changes and a reduction in friction between initially metallic surfaces.

A comparison of the tribological properties of WSe_x_/NP-W and WSe_x_S_y_/NP-W coatings revealed that moderate S doping does not significantly degrade the properties of these coatings in wet air but considerably enhances the performance in a N_2_-enriched environment at room temperature. At moderate doping levels characterized by (Se + S)/W < 3.5, sulfur atoms were dispersed throughout the matrix volume, potentially taking different states within the amorphous matrix packing. As a result, the hardness of the coating decreased, albeit the process being mitigated by the presence of W nanoparticles, promoting plastic deformation and densification of the WSe_x_S_y_/NP-W_3.6 coating under load. The introduction of S atoms appeared to alter the local atomic packing and may have contributed to the transformation of the amorphous structure into a crystalline/layered structure under tribo-impact. The partial substitution of Se atoms by S atoms in the layered structure is believed to have reduced the shear stresses between the basal planes in the 2H-W(Se/S)_2_ lattice. Notably, Hu et al. [35] have shown that TMD coatings containing a mixture of S and Se atoms exhibit superior tribological performance attributed to increased crystallinity and larger basal plane separations in these composites. To ensure the effective performance of WSe_x_S_y_/NP-W coatings under extremely challenging environmental conditions, such as very low temperatures and low humidity, the S content may need to be significantly increased, resulting in a much softer matrix.

W nanoparticles formed the hard “skeleton” of the WSe_x_/NP-W and WSe_x_S_y_/NP-W nanocomposite coatings. At relatively low counter-body loads, the nanoparticles in the coating could be removed from the surface without fracturing. Under higher loads, the particles underwent wear. However, the formation of a W(Se/S)_2_ tribofilm between the steel counter body and the W nanoparticle prevented direct contact between the metals, leading to a reduced friction coefficient. Notably, the contact between the counter body and the nanocomposite coating may have been localized primarily on the W nanoparticles, thus reducing the actual contact area and ultimately contributing to a lower friction coefficient.

A review of the literature on the tribological properties of WSe_x_ coatings synthesized by means other than PLD techniques reveals that the most comparable tribotesting conditions were achieved by Dominguez-Meister et al. [8,49,61], who employed nonreactive magnetron sputtering for WSe_x_ coating deposition. Reciprocating tests were conducted at 2 N of applied load (a maximum contact pressure of ~0.83 GPa), a stroke length of 2 mm, and a 2 mm s^−1^ of linear speed during 2500 cycles. The tribological behavior of pure WSe_x_ remains almost unaltered, showing a similar friction coefficient (0.04–0.07) and wear rates (1.5–3 × 10^−7^ mm^3^ N^−1^m^−1^), independent of the nature of the environment (in ambient air and in dry nitrogen). Mixing WSe_x_ with carbon or MoS_2_ did not enhance the low-friction properties [46,62]. However, nanocomposite WSe_x_S_y_/NP-W_3.6 coatings fabricated in this study by reactive PLD showed improved low-friction properties with a minimum friction coefficient of ~0.02, compared with the already known magnetron-deposited WSe_x_ coatings. The wear resistance of the WSe_x_S_y_/NP-W_3.6 coatings was not inferior to that of magnetron-deposited WSe_x_ coatings. The deposition of WSe_x_ coatings by magnetron sputtering has been extensively explored and optimized. However, the tribological performance of nanocomposite WSe_x_S_y_/NP-W coatings may be further improved by optimizing reactive PLD parameters.

## 5. Conclusions

This study has shown that subjecting the WSe_2_ target to pulsed laser ablation under vacuum conditions produces a flux of W and Se atoms, along with a flux of W nanoparticles. The maximum size of the nanoparticles can reach several tens of nanometers, although the structure of the coating is mostly made up of round-shaped particles smaller than 20 nm. The volume fraction of nanoparticles in the coatings can reach 25–30%. In the presence of H_2_S gas during laser ablation of WSe_2_ target, S atoms are introduced into the amorphous matrix of the coatings along with W and Se atoms, and their concentration increases with increasing H_2_S pressure. The incorporation of NP-W leads to an increased hardness of the nanocomposite coatings and may impact the contact area between the counter body and the coating.

Under moderate S atom concentration, WSe_x_S_y_/NP-W_3.6 coatings exhibit high-level tribological properties when tested in ambient air and N_2_-enriched environments at room temperature. The nature of tribo-induced changes in these coatings is contingent upon the load on the counter body. At higher counter-body loads, the coatings exhibited the lowest coefficient of friction (0.02) and the highest wear resistance (~1.6×10^−7^ mm^3^ N^−1^ m^−1^) when sliding in nitrogen. During sliding, an up to 20 nm-thick tribofilm forms on the coating surface containing W(Se/S)_2_ nanocrystals, whose basal planes align parallel to the coating surface. Importantly, the tribofilm effectively reduces the concentration of Se and S atoms to a value near stoichiometric ((Se + S)/W~1.9) and prevents W nanoparticles from making clean contact with the steel counter body. The tribological performance of these coatings, however, noticeably declines under more challenging conditions, such as low temperature (−100 °C) and N_2_-enriched environments with a relative humidity of ~7%.

Notably, the excessive S content in WSe_x_S_y_/NP-W_9 coatings has an adverse effect on their tribological properties under “conventional” ambient conditions. However, these coatings demonstrate the best tribological performance when tested at very low temperatures (about −100 °C), with a low wear rate and a coefficient of friction of 0.06 over 3000 sliding cycles. Other coatings experience a sharp increase in the coefficient of friction to 0.1 after just 1000 sliding cycles.

## Data Availability

Not applicable.

## Supplementary Materials

The following supporting information can be downloaded at https://www.mdpi.com/article/10.3390/nano13061122/s1, Figure S1: SEM images of (a) as-prepared and (b,c) laser-ablated WSe_2_ target with two magnifications; the area for EDS analysis is indicated in a red circle in (c); Figure S2: Distribution of elements in a local area of the WSe_2_ target subjected to laser ablation. The area is indicated in Figure S1c, and it contains spherical particles which were formed after laser ablation; Figure S3: XRD pattern of laser-ablated WSe_2_ target exhibits the content of (a) WSe_2_ (P6_3_/mmc), (b) α-W (Im3m), and (c) W_3_O/β-W (Pm3n) phases; Figure S4: Optical images of the wear tracks and wear scars for (a) WSe_x_/NP-W, (b) WSe_x_S_y_/NP-W_3.6, and (c) WSe_x_S_y_/NP-W_9 coatings after tribotesting in N_2_-enriched environment at a load of 5 N at −100 °C. The test duration for each coating is indicated in Figure 7.
