# Supplementary material for: Enhanced Tribological Performance of Low-Friction Nanocomposite WSexSy/NP-W Coatings Prepared by Reactive PLD
Source: Nanomaterials (Basel). 2023 Mar 21;13(6):1122. doi: 10.3390/nano13061122 (PMC10051849; doi:10.3390/nano13061122)
Supplement: Supplementary file 1 [file nanomaterials-13-01122-s001.zip › nanomaterials-2267426-supplementary.pdf]

Supplementary Materials

Enhanced Tribological Performance of Low-Friction Nanocomposite WSe_xS_y/NP-W Coatings Prepared by Reactive PLD

Vyacheslav Fominski [1,*], Dmitry Fominski [1], Maxim Demin [2], Roman Romanov [1] and Alexander Goikhman [2]

[1] National Research Nuclear University MEPhI (Moscow Engineering Physics Institute), Kashirskoe shosse. 31, 115409 Moscow, Russia
[2] Kant Baltic Federal University, A. Nevskogo St. 14, 236016 Kaliningrad, Russia
* Correspondence: vyfominskij@mephi.ru

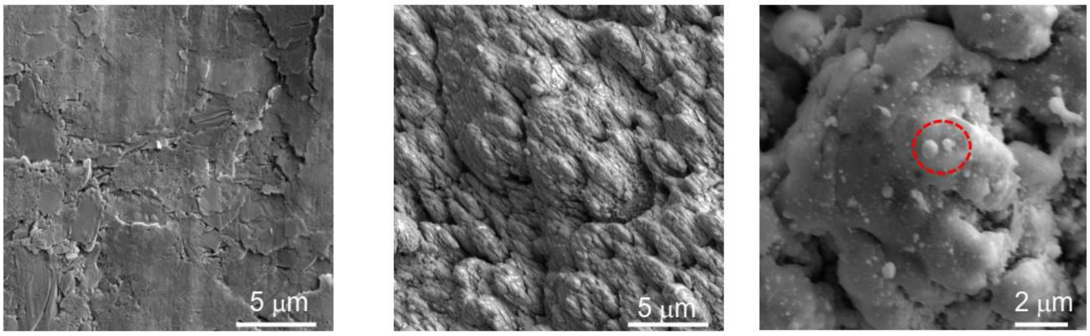

(**a**) (**b**) (**c**)

Figure S1. SEM images of (**a**) as prepared and (**b**,**c**) laser ablated WSe_2 target with two magnifications; the area for EDS analysis is indicated in a red circle in (**c**).

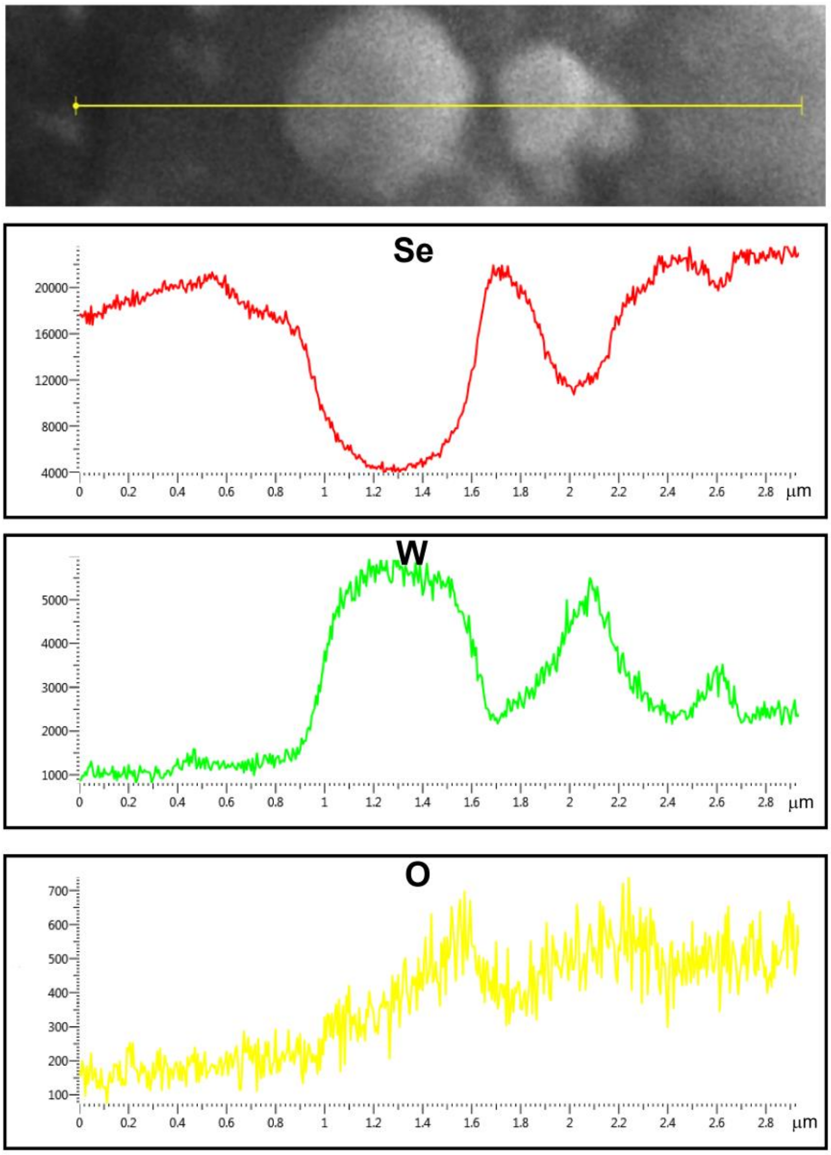

Figure S2. Distribution of elements in a local area of the WSe_2 target subjected to laser ablation. The area is indicated in Figure S1c, and it contains spherical particles which were formed after laser ablation.

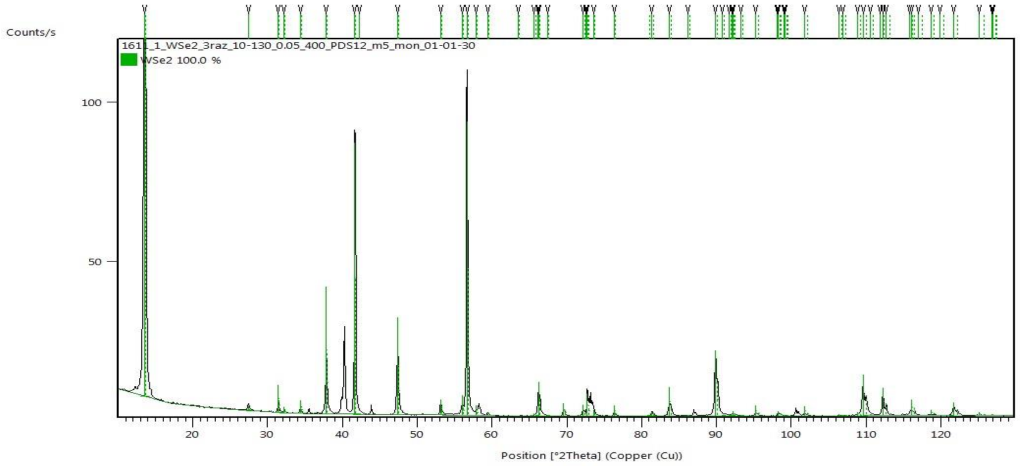

(**a**)

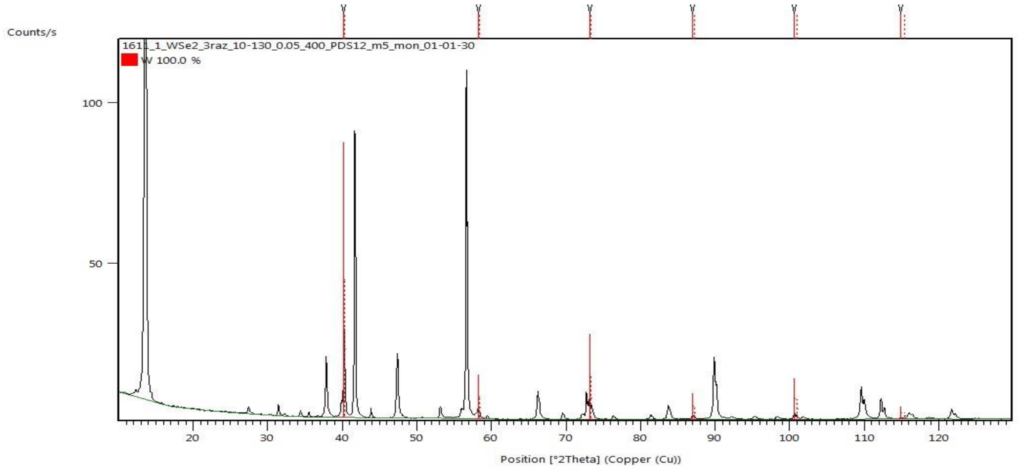

(**b**)

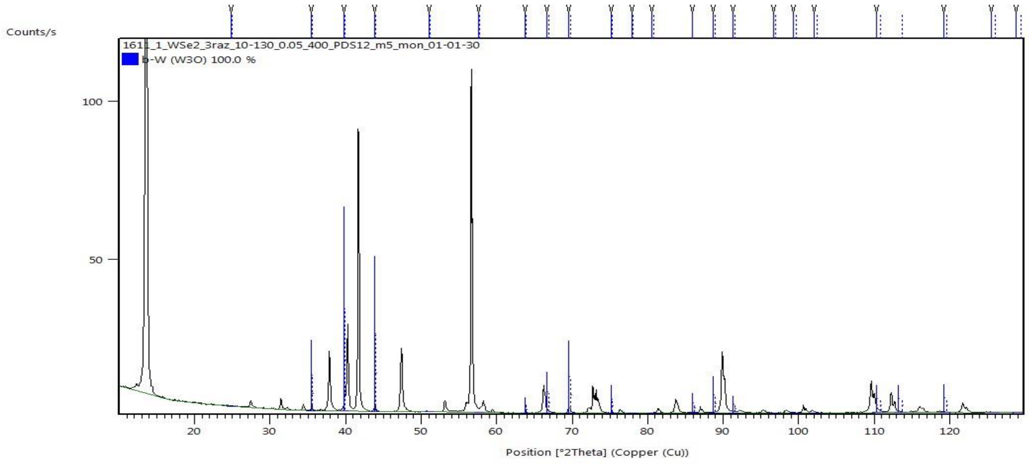

(**c**)

Figure S3. XRD pattern of laser ablated WSe_2 target exhibits the content of (**a**) WSe_2 ($P6_3/mmc$), (**b**) α-W (Im3m), and (**c**) W_3O/β-W (Pm3n) phases.

The diffraction patterns were measured on a PANalytical Empyrean Series 2 X-ray diffractometer using monochromatized CuKα radiation. The phase composition was analyzed using the PANalytical High Score Plus software and the ICCD PDF-2 database.

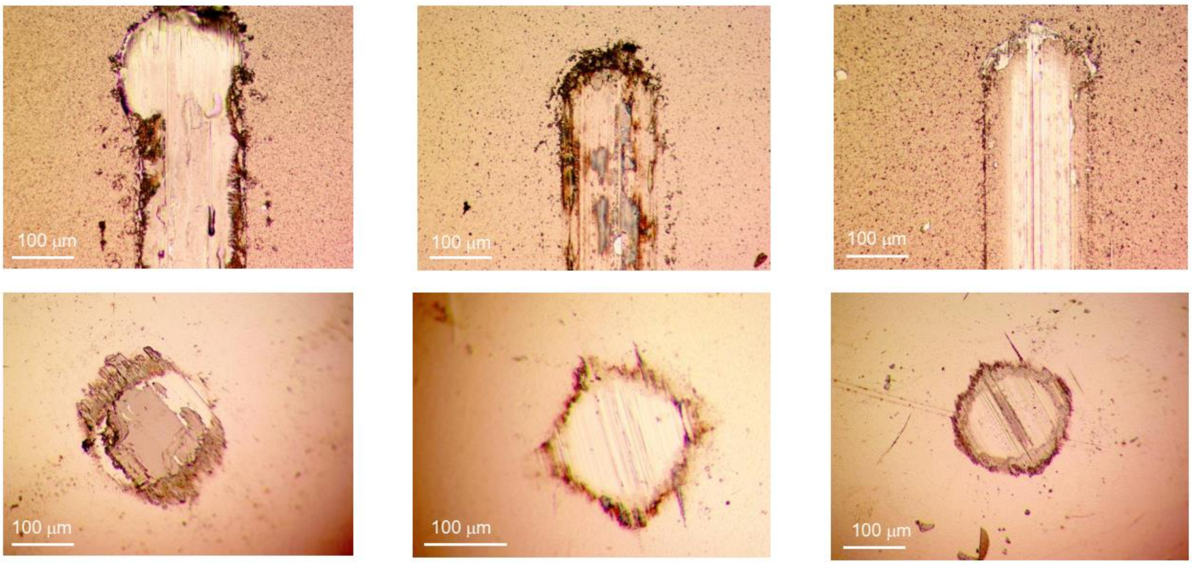

(**a**) (**b**) (**c**)

Figure S4. Optical images of the wear tracks and wear scars for (**a**) WSe_x/NP-W, (**b**) WSe_xS_y/NP-W_3.6, and (**c**) WSe_xS_y/NP-W_9 coatings after tribotesting in N_2-enriched environment at a load of 5 N at −100°C. The test duration for each coating is indicated in Figure 7.
